# Mapping out the structural changes of natural and pretreated plant cell wall surfaces by atomic force microscopy single molecular recognition imaging

**DOI:** 10.1186/1754-6834-6-147

**Published:** 2013-10-11

**Authors:** Mengmeng Zhang, Guojun Chen, Rajeev Kumar, Bingqian Xu

**Affiliations:** 1 Single Molecule Study Laboratory, College of Engineering and Nanoscale Science and Engineering Center, University of Georgia, Athens, GA 30602, USA; 2 Present address: Bruker Nano Surface Division, 112 Robin Hill Road, Santa Barbara, CA 93117, USA; 3 Center for Environmental Research and Technology, Bourns College of Engineering, University of California, Riverside, 1084 Columbia Avenue, Riverside, CA 92507, USA

**Keywords:** Plant cell wall, Dilute acid pretreatment (DAP), Surface structural changes, Carbohydrate-binding module, AFM recognition imaging, Recognition area percentage (RAP)

## Abstract

**Background:**

Enzymatic hydrolysis of lignocellulosic biomass (mainly plant cell walls) is a critical process for biofuel production. This process is greatly hindered by the natural complexity of plant cell walls and limited accessibility of surface cellulose by enzymes. Little is known about the plant cell wall structural and molecular level component changes after pretreatments, especially on the outer surface. Therefore, a more profound understanding of surface cellulose distributions before and after pretreatments at single-molecule level is in great need. In this study, we determined the structural changes, specifically on crystalline cellulose, of natural, dilute sulfuric acid pretreated and delignified cell wall surfaces of poplar, switchgrass, and corn stover using single molecular atomic force microscopy (AFM) recognition imaging.

**Results:**

The AFM tip was first functionalized by a family 3 carbohydrate-binding module (CBM3a) (*Clostridium thermocellum* Scaffoldin) which specifically recognizes crystalline cellulose by selectively binding to it. The surface structural changes were studied at single molecule level based on the recognition area percentage (RAP) of exposed crystalline cellulose over the imaged cell wall surface. Our results show that the cell wall surface crystalline cellulose coverage increased from 17-20% to 18-40% after dilute acid pretreatment at 135°C under different acid concentrations and reached to 40-70% after delignification. Pretreated with 0.5% sulfuric acid, the crystalline cellulose surface distributions of 23% on poplar, 28% on switchgrass and, 38% on corn stover were determined as an optimized result. Corn stover cell walls also show less recalcitrance due to more effective pretreatments and delignification compared to poplar and switchgrass.

**Conclusions:**

The dilute acid pretreatment can effectively increase the cellulose accessibility on plant cell wall surfaces. The optimal acid concentration was determined to be 0.5% acid at 135°C, especially for corn stover. This study provides a better understanding of surface structural changes after pretreatment such as lignin relocation, re-precipitation, and crystalline cellulose distribution, and can lead to potential improvements of biomass pretreatment.

## Background

The fossil fuel scarcity has become a serious problem and exploring alternative energy sources has drawn increasing attentions from both academia and industry. To find renewable and sustainable replacements of crude oil, the lignocellulosic biomass (such as poplar, switchgrass, and corn stover), has been considered as one of the primary feedstocks with potentials of high efficiency and low cost [1-4]. Enzymatic hydrolysis of plant cell walls has overwhelming advantages over chemical treatments of lower energy consumption, less hazardous by-products, nearly theoretical yields, etc. [3,5]. As the most promising strategy, direct hydrolysis of plant cell wall cellulose (the major component of plant cell walls) by enzymes, however, is greatly hindered due to the natural complexity of the plant cell walls [1,6,7]. The cellulose microfibrils are embedded in the cross-linked hemicellulose and lignin matrix, which reduces the direct accessibility of enzyme binding to them [7-9]. To overcome this recalcitrance, several pretreatments have been developed to enhance the cellulose degradability [10-12].

The essential role of pretreatment is to physically and/or chemically disassemble the protective carbohydrate-lignin complex, disrupt the crystalline structure of cellulose and more importantly, increase the surface accessibility of plant cell wall carbohydrates [11,13]. Some extensively studied pretreatment technologies include steam explosion [14], ammonia fiber expansion (AFEX) [15], ammonia recycled percolation (ARP) [16], lime [17], dilute acid pretreatment [18], etc. Each pretreatment has specific advantages and disadvantages in hemicellulose degradation and lignin removal, but all have been proven to change the plant cell wall structure [10]. Dilute acid pretreatment (DAP), especially with dilute sulfuric acid, has received extensive attentions for several decades in fuel production. Its major objective is to extensively solubilize hemicellulose (over 80% of the natural content) and disrupt the carbohydrate-lignin linkage to enhance the enzymatic digestibility of cellulose [11,19,20]. Although little lignin is removed, the disruption and re-localization of lignin have been clearly verified which can slightly increase the exposed surface area of cellulose for hydrolysis [21-23].

Enzymatic hydrolysis usually proceeds from outer surface of the plant cell wall. Extensive distribution of hydrolysable components (i.e., cellulose and hemicellulose) on the surface therefore can facilitate the cell wall-enzyme interactions and improve the hydrolysis efficiency. Consequently, an in-depth understanding of structural changes of pretreated cell wall surface, especially at single molecule level, can provide a fundamental insight of cell wall ultrastructure for pretreatment improvement. Some frequently used techniques to characterize the plant cell walls are X-ray diffraction (XRD), nuclear magnetic resonance (NMR), infrared spectroscopy (IR), high performance liquid chromatography (HPLC) [10,24-26], etc. These techniques are usually used as a combination to identify the structural and component changes of the plant cell walls. However, the plant cell wall structure following pretreatments is completely or partially destroyed and the chemical changes determined by bulk chemical analysis do not necessarily reflect the changes on the surface. Alternatively, scanning electron microscopy (SEM), transmission electron microscopy (TEM), and atomic force microscopy (AFM) can provide surface morphology information but do not provide information on chemical composition [25,27,28]. Fluorescence microscopy [29] and time-of-flight secondary ion mass spectrometry (ToF-SIMS) [30] are also applied in cell wall surface analysis, but the accuracy of component distributions on surface is unsatisfactory due to the limited mapping resolution.

AFM recognition imaging by functionalized AFM tips has been widely used to map the substrate components at single molecule level [31,32]. The method is based on detecting small shift in the peak value of the cantilever deflection signal that occur when a tip-tethered molecule (CBM) binds to a target (cellulose) on the surface, bridging the gap between the surface and an oscillating tip [32]. When combined with the single molecule dynamic force spectroscopy (SMDFS), this technique is capable of measuring unbinding forces and dynamic and kinetic parameters of the specific interactions between the molecules functionalized on the AFM tip and those immobilized on flat substrate, even on living cells [33,34]. The AFM recognition imaging is also used as a reliable and efficient tool in studying carbohydrate-protein interactions [35]. Recently, AFM recognition imaging and SMDFS have been applied to map the natural and pretreated poplar cell wall surface and study the affinity between a non-catalytic family 3 carbohydrate-binding module (CBM3a) and crystalline cellulose [36,37]. The crystalline cellulose exposed on cell wall surface was specifically recognized and its surface coverage was quantified [37].

In this study, we imaged the natural, dilute sulfuric acid pretreated and delignified plant cell wall surfaces of poplar, switchgrass, and corn stover by AFM recognition imaging. The AFM tip was functionalized by crystalline cellulose-binding CBM3a molecules (derived from *Clostridium thermocellum* Scaffoldin CipA). The surface structural changes of plant cell walls before and after pretreatments were measured and compared based on the recognition area percentage (RAP) of exposed crystalline cellulose.

## Results and discussion

### Principles of AFM recognition imaging

**Figure 1:**
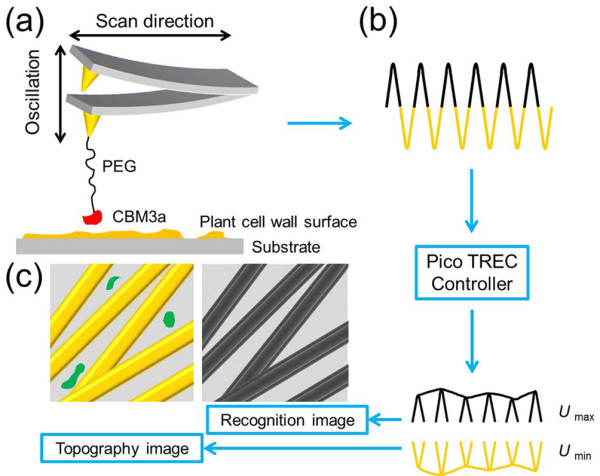
**Schematics of AFM recognition imaging. (a)** AFM tip modification and recognition imaging process; **(b)** topography and recognition signal division by PicoTREC controller; **(c)** example of topography and recognition images generated by separated signals. Yellow strips: sketch of crystalline cellulose; black strips: recognition signal of crystalline cellulose in topography image; green marks: other components which do not have specific interactions with functionalized AFM tip and show no recognition signals in the recognition image.

The crystalline cellulose distributions on natural and pretreated plant cell wall surfaces were specifically characterized by the well-established recognition imaging technique (Figure 1). The AFM tip was first coated with a magnetic material followed by a thin gold layer. The pre-coated tip was then functionalized by thiol-PEG_2000_-NTA-Ni crosslinker in water and CBM3a molecules in Tris-Cl buffer [36]. The long and flexible PEG_2000_ was a commonly used crosslinker for minimizing the steric hindrance and misorientation [38,39]. Here, the active residues involved in crystalline cellulose binding are located at the “flat bottom” of the CBM3a molecule on the opposite side of the location of (His)_6_-tag, providing enough freedom for binding [40,41]. The gold-NTA-Ni-(His)_6_-tagged protein has been widely used as a stable chelating complex for specific binding [42].

Figure 1(a) shows a schematic of a modified AFM tip imaging the plant cell wall surface. When the crystalline cellulose is bound by CBM3a, the crosslinker will be stretched in the retraction process of the AFM cantilever. The top peak of the oscillations is reduced due to this energy loss and the specific interactions can be detected by generating a corresponding recognition signal. This process is followed by a further analysis in PicoTREC controller, which can split the raw deflection signal of the cantilever into the upper (*U*_*max*_, marked in black) and lower (*U*_*min*_, marked in yellow) parts. These two parts of each circle are then recorded as the recognition and topography signals, respectively (Figure 1(b)) [41]. The pattern formed by several dark recognition patches clearly coincide with the positions of the crystalline cellulose in the topography image; meanwhile, the molecule on the substrate which doesn’t have specific interactions with the modified AFM tip will not generate recognition signal, i.e., the green marks shown in the topography image in Figure 1(c). This recognition process has been widely used due to its great potential in molecular imaging of surfaces, while such measurements are very tedious and subject to errors and therefore great caution should be taken during sample preparation, data acquisition and interpretation [31,32]. For example, proper concentrations of CBM are critical to ensure single or just a few CBM molecules be modified on the AFM tip to avoid multi-molecular interactions.

### Surface mapping of natural plant cell walls of poplar, switchgrass, and corn stover

**Figure 2:**
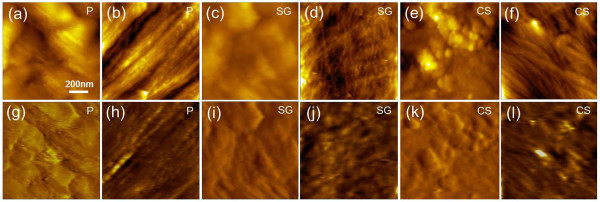
**AFM topography and recognition images of natural plant cell walls.** Topography **(a-f)** and recognition **(g-l)** images of natural poplar (P), switchgrass (SG), and corn stover (CS). **(a, c, e, g, i, k)** show the representative surface area mainly covered by lignin and **(b, d, f, h, j, l)** show the representative surface area mainly covered by crystalline cellulose.

Previously, we clearly visualized and recognized the crystalline cellulose on the surface of natural poplar slice. The recognition signal comes from the interaction between the CBM-functionalized AFM tip and the molecules on the sample surface. Therefore, to prove the specificity of the recognition, we measured the unbinding forces between the AFM tip (both CBM3a- functionalized and bare tip) and the poplar slice surface. The results indicated a pronounced, specific unbinding force peak in the force histograms for CBM3a-crystalline cellulose interaction, while near-zero, non-specific force was obtained when taking the force data for all the non- cellulose areas on the poplar slice surface. For bare AFM tip, only the near-zero, non-specific force was observed for all the areas on the poplar slice surface [36,37]. In this work, we also characterized the natural cell wall of switchgrass and corn stover and the representative topography and recognition images are shown in Figure 2.

It has been confirmed that the lignin content in poplar is about 10% higher than that in switchgrass and corn stover [25,27]. Accordingly, we observed more surface area covered by lignin or lignin-carbohydrate complex on the poplar plant cell wall than on switchgrass and corn stover. Besides, the morphology of poplar lignin exhibited smooth and intact layers while the lignin in switchgrass and corn stover formed irregular and compact granules as shown in the topography images of Figure 2(a), (c), and (e). Few surface components of these areas were recognized in the corresponding recognition images of Figure 2(g), (i), and (k), indicating the absence of specific interactions between non-cellulose components and CBM3a molecule on the AFM tip. Differently, on surface area extensively covered by parallel or interwoven crystalline cellulose microfibrils in Figure 2(b), (d), and (f), strong recognition signals were detected in the corresponding recognition images of Figure 2(h), (j), and (l).

### Measurement of recognition area percentage (RAP) on plant cell wall surfaces

The crystalline cellulose distributions on the plant cell wall surface were quantitatively determined based on recognition signal distribution. For each biomass sample, 5 different surface areas in average were imaged on each single piece by the functionalized AFM tip. Over 20 sample pieces were imaged and 100 recognition images in size of 1 μm × 1 μm were randomly selected for the RAP calculation. Generally, all the recognition images of each sample were divided into maximum 7 types based on the surface features represented by RAP of crystalline cellulose. Table 1 lists a summary of RAPs of each area type on pretreated and delignified corn stover cell wall surface (sample named as 0.5%CS-135 with 0.5% sulfuric acid concentration pretreated at 135°C). The details of definition and calculation of area types are given in the Additional file 1: Section 2.

**Table 1:** Recognition area percentage calculation of 0.5%CS-135 and delignified 0.5%CS-135 surface (100 images)

**Sample ID**	**Area type**	**Image counts**	**Average RAP (%)**	**Total RAP (%)**
0.5%CS -135	A	6	7.15	27.9
	B	19	17.0	
	C	25	24.7	
	D	28	34.9	
	E	9	44.8	
	F	3	58.5	
	G	0	-	
0.5%CS -135 delignified	A	0	0	42.6
	B	0	0	
	C	12	26.7	
	D	21	34.5	
	E	36	43.1	
	F	25	52.4	
	G	6	59.5	

Type A: < 10%; B: 10-20%; C: 20-30%; D: 30-40%; E: 40-50%; F: 50–60; G: > 60%.

**Figure 3:**
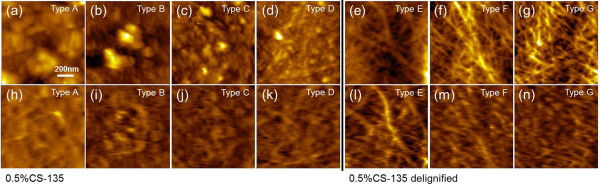
**Representative topography and recognition images of 0.5%CS-135 and delignified 0.5%CS-135 surface of different area types. (a-g)** topography images; **(h-n)** recognition images. 0.5%CS-135, corn stover cell wall pretreated by 0.5% sulfuric acid at 135°C.

The representative topography and recognition images selected from each area type are shown in Figure 3. Based on surface components of pretreated cell wall before and after delignification, the majority of recognition images of 0.5%CS-135 were classified into type A through D, while most of the recognition images of delignified 0.5%CS-135 showed the features of type E-G. The type A surface was mainly covered by irregularly shaped agglomerates with different sizes, which were mainly supposed to be the re-localized lignin after DAP [23]. The RAP of Figure 3(h) was lower than 10%, indicating little CBM3a-cellulose interactions. From Type B to G, the amount and size of surface agglomerates gradually decreased and more crystalline cellulose appeared, resulting in an increase of RAPs to over 60%. This difference denoted that the lignin locating on the surface of pretreated plant cell walls was extensively removed during the delignification process, therefore the crystalline cellulose underneath was exposed and recognized by the CBM3a-modified AFM tip [28].

### Effect of dilute acid pretreatment under different concentrations

**Figure 4:**
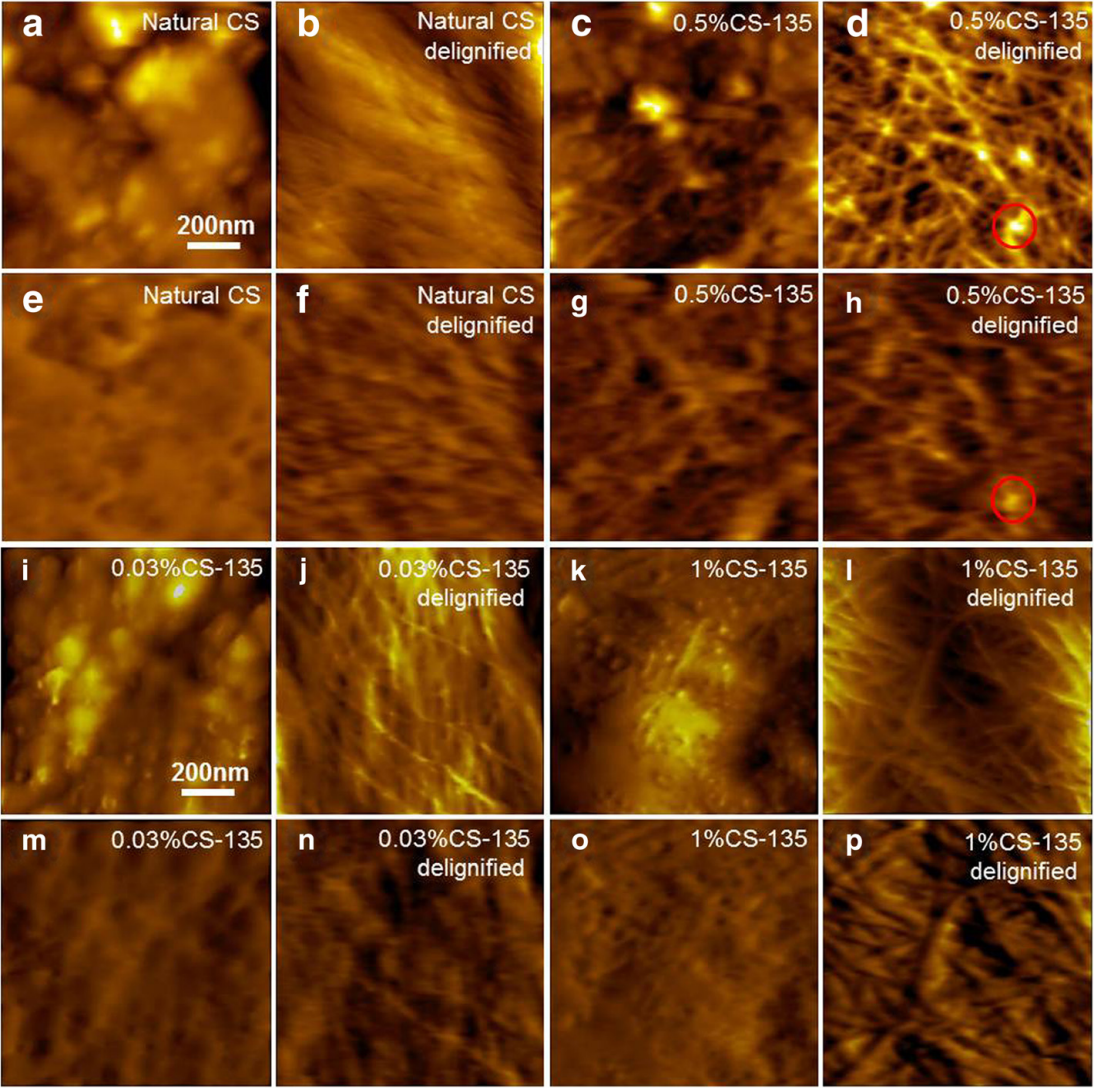
**Topography and recognition images of natural, dilute acid pretreated, and delignified corn stover cell wall.** Topography **(a-d, i-l)** and recognition **(e-h, m-p)** images of natural, dilute acid pretreated (pretreated by 0.03%, 0.5%, and 1% sulfuric acid at 135°C) and delignified corn stover cell wall. Some residues in **(d)** which do not have recognitions in **(h)** are highlighted in the red circles.

Hydrolysis of hemicellulose is considered as the main reaction occurring during acid pretreatment accompanied by fast condensation and precipitation of solubilized lignin as inevitable physical process [10,43]. The coalesced lignin deposits back onto the plant cell wall surfaces and potentially block further access to cell wall components as observed by SEM and AFM [23,44]. To quantitatively determine the component changes before and after DAP, we compared the surface of natural, dilute acid pretreated and delignified plant cell walls and calculated the RAPs. The plant cell wall surfaces were predominantly covered by lignin sheath, which was unevenly distributed all over the surface (Figure 4(a)). Recognition signals can hardly be seen due to the absence of specific CBM3a-cellulose interactions (Figure 4(e)). Remarkably, more crystalline cellulose microfibrils could be seen on the surface of 0.5%CS-135 sample in Figures 4(c) and (g) with higher RAPs than that of poplar (0.5%P-135) and switchgrass (0.5%SG-135) (Additional file 1: Section 3.1). This result demonstrated that the cell wall of corn stover is more vulnerable to DAP process.

To determine the effect of DAP on cell wall surface structural changes other than lignin re-distribution, we removed the lignin by acidified sodium chlorite as shown in Figure 4(b, d, f and h). In Figure 4(b), the crystalline cellulose exhibited a compact configuration and individual crystalline cellulose microfibrils could hardly be distinguished. Some agglomerates could also be observed in Figure 4(d), which were supposed to be lignin residues (marked in red circles) that could not be recognized in Figure 4(h). The RAPs of natural and delignified corn stover were measured to be 15.2% and 29.4%, respectively. The RAPs of natural corn stover and switchgrass (RAPs of natural switchgrass increased from 13.6% to 26.1% after delignification) nearly doubled after delignification; meanwhile the RAPs of delignified poplar increased by 70% (RAPs of natural poplar increased from 12.9% to 21.8% after delignification) (Additional file 1: Section 3.1 and 3.2). The less efficient delignification effect on poplar might due to a naturally higher content of intact lignin and a more solid cell wall structure. Comparatively, the surfaces of delignified cell walls after 0.5% sulfuric acid pretreatment at 135°C exhibit a more interrupted and interwoven configuration, especially for corn stover (Figure 4(d)) and switchgrass (Additional file 1: Section 3.2 Figure S3(d)). This morphology change was mainly caused by the removal of hemicellulose, therefore the linkage between crystalline cellulose was destroyed and individual crystalline cellulose microfibrils were released. The RAPs of 0.5%CS-135, 0.5%SG-135 and 0.5%P-135 were 27.9%, 20.4%, and 17.4%, respectively. After delignification, the RAPs of dilute acid pretreated corn stover and switchgrass increased by over 50% of their pretreated condition and that of pretreated poplar even doubled (Additional file 1: Section 3.2). The higher cellulose content in natural plant cell walls of poplar also contributed to a more substantial increase of RAP when more re-localized lignin droplets was removed (Additional file 1: Section 3.2 Figure S3(f)).

Various DAP conditions (e.g., altering temperature, acid concentration, reaction time, etc.) have been investigated to optimize the pretreatment results for better cell wall degradability [24]. The hemicellulose removal and re-localization of lignin are proven to be more pronounced with stronger acid or higher reaction temperature [10,23]. In the following work, we quantitatively studied the surface structural changes during DAP under different acid concentrations (i.e., 0.03%, 0.5%, 1%, and 2%) as shown in Figure 4(i-p). The surface structural changes after 0.03% and 1% DAP can be clearly seen in Figure 4(i and k). The size of agglomerates formed on the cell wall surfaces after 0.03% DAP was larger than that observed on surfaces pretreated by 1% acid, especially on 0.03%P-135 and 0.03%SG-135 (Additional file 1: Section 3.3 Figure S4). The re-localized lignin was supposed to overlay the cell wall surfaces more evenly under high acid concentration and thereby to some extent, reduce the RAPs [23]. Some hemicellulose re-precipitated onto the cell wall surfaces under lower acid concentrations was also supposed to decrease the RAPs [22]. The same trend could also be observed on cell wall surfaces pretreated by 2% acid (Additional file 1: Section 3.5, Figure S6(a, c and e)).

To further understand the effect of acid concentrations on hemicellulose removal, we also removed the surface lignin to see the compositional changes underneath. In Figure 4(j), the exposed crystalline cellulose on 0.03%CS-135 presented some interwoven arrangement, similar to the morphology observed on 0.03%SG-135 (Additional file 1: Section 3.4 Figure S5(c)); while the exposed crystalline cellulose exhibits a more intact, parallel structure on 0.03%P-135 (Additional file 1: Section 3.4 Figure S5(a)). Moreover, less surface components were recognized on 0.03%P-135 (Additional file 1: Section 3.4 Figure S5(e)) than on 0.03%SG-135 (Additional file 1: Section 3.4 Figure S4(g)) and 0.03%CS-135 in Figure 4(n). Therefore, the surface of delignified 0.03%P-135 was supposed to be covered by a large amount of hemicellulose, denoting a less effective removal of this component. When the cell walls of corn stover were previously pretreated with stronger acid, e.g., 1% or 2% acid, a denser, more regular crystalline cellulose structure were exposed after delignification (Figure 4(l)) and the corresponding recognition area also greatly increased (Figure 4(p)). The similar results were also observed on the delignified poplar and switchgrass (Additional file 1: Section 3.4 Figure S5(b and d)). The images of cell walls pretreated by 2% sulfuric acid at 135°C are shown and discussed in Additional file 1: Section 3.5 Figure S6.

Similarly, the RAPs of 0.03%CS-135 and 1%CS-135 were determined to be 19.4% and 15.9%, respectively. The RAPs of 0.03%P-135 and 0.03%SG-135 also decreased from 14.8% to 13.5% and 17.3% to 15.2%, respectively when compared with 1%P-135 and 1%SG-135. The results demonstrated that the RAPs decreased with a higher acid concentration which caused more extensive and even lignin coverage by smaller lignin droplets and corn stover exhibited better accessibility. After delignification, the RAP of 0.03%CS-135 was 35.3% and the RAP of 1%CS-135 became 50.8%. The RAPs of delignified poplar and switchgrass also showed the same trend, i.e., the RAPs of poplar increased from 29.4% (0.03%P-135) to 41.2% (1%P-135), while the RAPs of switchgrass increased from 31.1% (0.03%SG-135) to 43.8% (1%SG-135) (Additional file 1: Section 3.4). Hence, compared with 0.03% acid, cell wall pretreatment with 1% acid exhibited 6-12% more surface accessibility of crystalline cellulose by dissolving more hemicellulose, although the surface lignin was hardly removed. Notably, the polymerization of the crystalline cellulose was also affected under the high acid concentration by inducing the decrease of microfibril length as shown in Figure 4(d and l), therefore more reducing-ends were supposed to be produced (Additional file 1: Section 3.3 and 3.4).

To sum up, the dilute acid pretreatment effectively dissolve hemicellulose but showed no pronounced removal of lignin, at least on the outer surface of plant cell wall. The plant cell wall was also deconstructed due to removal of hemicellulose and lignin after delignification. The corn stover cell wall showed less recalcitrance compared to poplar and switchgrass.

### Area type distributions and RAP summary of natural and pretreated plant cell walls

**Figure 5:**
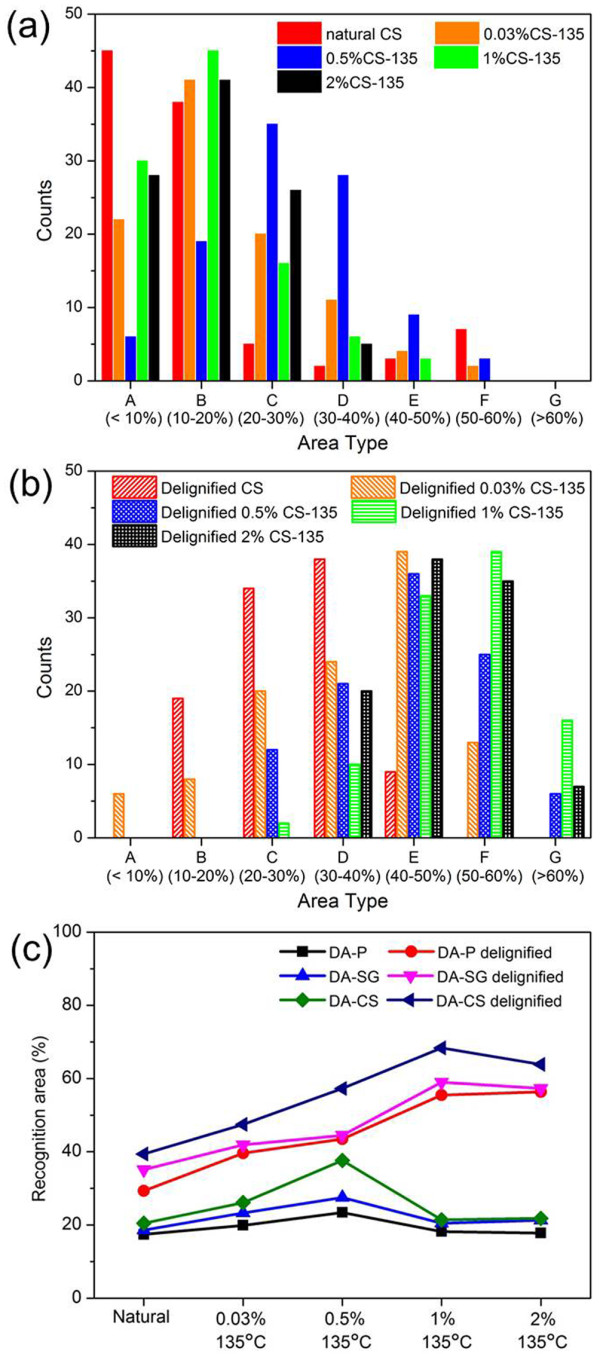
**Area type distributions and recognition are percentage summary. (a)** Area type distributions of natural and dilute acid pretreated cell wall surfaces of corn stover; **(b)** Area type distributions of delignified cell wall surfaces of corn stover; **(c)** Recognition area percentage (RAP) summary of natural and pretreated poplar, switchgrass, and corn stover cell wall surfaces.

As an average value, however, RAP cannot tell the differences of surface conditions before and after pretreatments. For instance, the RAP of natural corn stover is 15.2%, which is very close to the RAPs of 1%CS-135 (15.9%) and 2%CS-135 (16.2%). However, it doesn’t necessarily mean that the surface component distributions are the same. To explore the changes of plant cell wall structures, the number of recognition images in each area type was counted and the results were compiled in Figure 5.

The distributions in Figure 5(a) clearly manifested that for natural corn stover, more than half of the surface structures were defined as type A and B and about one fourth of the recognitions images showed the features of type E and F. The crystalline cellulose in natural plant cell walls were highly ordered, lacking the favorable position for specific CBM binding, therefore the recognized area was quite limited and the RAP cannot reach to over 60% as defined in type G. After DAP, over 60% of surface features were grouped in type B and C, indicating a slight interruption of lignin coverage and higher crystalline cellulose accessibility. Remarkably, for 0.5%CS-135, the majority of recognition images located in type C and D, which also gave a much higher RAP value and exhibited a more evenly distributed crystalline cellulose.

After removal of lignin, the image distributions changed evidently as shown in Figure 5(b). The area types distributed more widely and more surface features were determined as type D through G. For delignified corn stover without DAP, more than half of the images areas revealed features of type C and D, whereas the majority of surface features of 0.03%CS-135, 1%CS-135, and 2%CS-135 were determined as type D through F. Some areas classified as type G, especially on 2%CS-135, validated a more effective removal of hemicellulose under higher acid concentration. Similar results could also be obtained on poplar and switchgrass as shown in Additional file 1: Section 4.

Due to the limitation of high scan speed during imaging, surface roughness and unfavorable position of crystalline cellulose for binding, the recognition efficiency and accuracy were supposed to have their own limitations [32,45]. Therefore, to reflect more accurate surface crystalline cellulose distributions, we used Avicel as a control to calibrate the RAPs. The RAP of Avicel, a commercial product containing up to 97% of microcrystalline cellulose, was measured to be 72.1% (Additional file 1: data Section 5). Hence the recognition efficiency was simply defined as 72.1% / 97% = 74.3%. After calibration, we summarized the new RAPs obtained under different acid concentrations at 135°C into Figure 5(c).

Figure 5(c) illustrates a direct view of RAPs measured on all biomass species under different pretreatment conditions. After DAP, the RAPs of poplar, switchgrass, and corn stover increased slightly and reached a highest value when the acid concentration was 0.5% and then decreased. At the acid concentration higher than 1%, the surface structure was supposed to have less correlation to the acid concentration and no further improvement of RAPs was observed. With 0.5% DAP, the cell wall deconstruction, hemicellulose removal, and lignin re-precipitation reached to a balance point so that the crystalline cellulose skeleton was exposed to a maximum level with the highest RAP. After delignification, however, the RAPs could be correlated to acid concentration up to 2%. When the surface lignin droplets were nearly completely removed, the blocking effect was greatly eliminated and the cell walls pretreated under higher acid concentrations presented more interrupted structures with more binding-favorable positions of crystalline cellulose. A slight decrease of RAPs in switchgrass and corn stover pretreated by 2% acid could be attributed to a more evident effect of depolymerization of crystalline cellulose, lower surface density of surface crystalline cellulose microfibrils due to extensive removal of hemicellulose or partial generation and deposition of pseudo-lignin [46,47].

In summary, the RAPs after calibration indicated that the increasing acid concentration caused more effective removal of hemicellulose on all plant cell wall surfaces; meanwhile the surface coverage of re-precipitated lignin droplets reduced slightly and then intensified with highest RAP at 0.5% acid. The surface chemical components changes were further qualitatively determined by grazing angle attenuated total reflectance Fourier transform infrared (ATR-FTIR) spectroscopy. The spectra (poplar, as an example) indicated a conclusion similar to RAP results (Additional file 1: Section 6, Figure S9).

### Effect of dilute acid pretreatment under different temperatures

**Figure 6:**
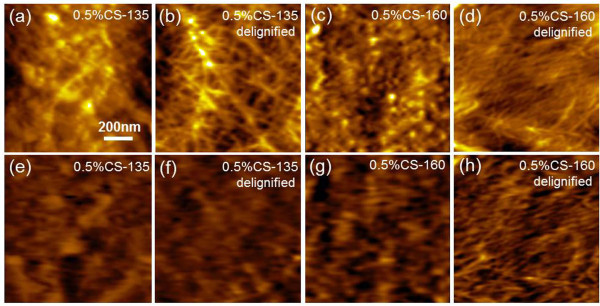
**Topography and recognition images of dilute acid pretreated (135°C and 160°C) and delignified corn stover cell wall. (a-d)** Topography images; **(e-h)** Recognition images. 0.5%CS-135, corn stover cell wall pretreated by 0.5% sulfuric acid at 135°C; 0.5%CS-160, corn stover cell wall pretreated by 0.5% sulfuric acid at 160°C.

DAP under high temperatures (130°C-220°C) was considered to highly improve plant cell wall deconstruction [23]. According to Moxley *et al*. [22], cellulose accessibility was closely correlated to hemicellulose solubilization at lower pretreatment temperatures; at higher temperatures, however, lignin degradation has a better correlation with cellulose accessibility. Here we compared the surface structural changes of corn stover and switchgrass cell walls pretreated by 0.5% acid at 135°C and 160°C. Figure 6(a, c) reveal re-deposited lignin droplets on the cell wall surfaces. Notably, the droplet on surfaces of 0.5%CS-135 was slightly larger than that on surfaces of 0.5%CS-160. This difference could be attributed to depolymerization and translocation of lignin under higher pretreatment temperature [23].

After delignification, the deconstructed cell wall surfaces were fully exposed with features of parallel and interwoven crystalline cellulose. As shown in Figure 6(b and d) and Additional file 1: Section 6 (Figure S9), the crystalline cellulose microfibrils on surfaces of 0.5%CS-160 and 0.5%SG-160 were better separated than those on surfaces of 0.5%CS-135 and 0.5%SG-135, depicting a more in-depth and delicate removal of hemicellulose among individual cellulose microfibrils. The recognition signals in Figure 6(h) also showed more delicate distribution compared with that in Figure 6(f). On the other hand, 0.5%SG-135 and 0.5%CS-135 showed the RAPs of 27.5% and 37.6%, respectively (after calibration, the same for the following RAPs). These values were slightly lower than the RAPs of 0.5%SG-160 (28.5%) and 0.5%CS-160 (39.9%). Corn stover cell walls again seemed to be more sensitive to higher temperature. Accordingly, the RAPs of delignified 0.5%SG-135 and 0.5%CS-135 were measured to be 44.5% and 57.3%, respectively, lower than the RAPs of 0.5%SG-160 (47.8%) and 0.5%CS-160 (61.1%) respectively. Therefore, DAP under higher temperature revealed a higher efficiency in plant cell wall deconstruction, especially for corn stover.

## Conclusions

Based on AFM recognition imaging and area percentage calculations, our results showed that 17-20% of plant cell wall surfaces were covered by crystalline cellulose before pretreatment and this coverage increased to 23-38% after dilute acid pretreatment under different temperature and acid concentrations. When the plant cell walls were pretreated with 0.5% sulfuric acid, the crystalline cellulose surface distribution of 23% on poplar, 28% on switchgrass, and 38% on corn stover was determined as an optimized result at 135°C. Compared to bulk component analysis, this method exhibits pronounced advantages in providing detailed surface information of plant cell walls.

## Methods

### Preparation of recombinant CBM3a and AFM tip functionalization

The recombinant CBM3a was provided by Complex Carbohydrate Research Center, University of Georgia. The AFM tips used (CS-10 silicon) were purchased from Nanoscience Instruments, Phoenix, AZ with the nominal spring constant of 0.1 N/m. The preparation method of recombinant CBM3a and the CBM3a-AFM tip functionalization procedure have been described in details elsewhere [36].

### Dilute sulfuric acid pretreatment of biomass samples

All biomass for pretreatment were ball-milled (8000 M Mixer/Mill, SPEX SamplePrep, Metuchen NJ) and sieved by mesh screen. The sample pieces in size of 200–250 μm were collected, washed by DI water, and dried at 45°C for 24 h. 0.1 g ball-milled biomass of each species was pre-soaked in dilute sulfuric acid (0.03% w/w, 2 mL) (VWR, Radnor, PA) for 30 min in a 20 mL glass pressure tube (Ace Glass Incorporated, Vineland, NJ). The sealed pressure tube was heated in a heating block on a hot plate (Barnstead/Thermolyne – RT Elite, Dubuque, IA) at 135°C for 20 min. The reaction was stopped by cooling down the tube to room temperature in cold DI water. The pretreated sample was then washed by 10 mL DI water for 5 times and centrifuged with 1 mL DI water (5,000 rpm, 10 min) for 5 times (SORVALL BioFuge Pico Microcentrifuge, Thermo Electron Corporation, Waltham, MA) and finally dried in air at 45°C for 24 h. The same procedure was repeated at 135°C for all three species with 0.5% w/w, 1% w/w, and 2% w/w sulfuric acid. The switchgrass and corn stover pretreated in 0.5% w/w sulfuric acid at 160°C (0.095 g H_2_SO_4_: dry wt, 5 wt.% solids) were provided by the University of California at Riverside. The summary of all pretreatment conditions and sample nominations are compiled in Table 1 in the Additional file 1: Section 1.

### Delignification of dilute acid pretreated biomass samples

The pretreated samples were subsequently delignified following one of the leading methods [28]. Briefly, 0.02 g sodium chlorite (J. T. Baker, Phillipsburg, NJ) and 40 μL glacial acetic acid (VWR, Radnor, PA) was added into each pretreated biomass water slurry (3% solid, 1 mL). The reaction was taken at 80°C for 1.5 h with gentle stirring. After cooling down, the bleached sample was washed 8 times with DI water followed by centrifugation at 10,000 rpm for 10 min and then was dried in air at 45°C for 24 h.

### AFM sample preparation and experimental setup for recognition imaging

The AFM sample preparation procedure was the same as poplar slice immobilization as described in our previous paper [36]. The PicoPlus Molecular Imaging system together with a PicoScan 3000 Controller was used in this work. An Agilent multipurpose AFM scanner with open loop was used for all recognition imaging. All images were taken using non-contact, top magnetic AC (TopMAC) mode under PicoTREC (Agilent Technologies, Santa Clara, CA). The topography (height) and recognition images were conducted simultaneously [32,37]. The 200–500 μm hand-cut, natural pieces of each biomass sample after mesh screening (200–500 μm in size) before pretreatments and pure microcrystalline cellulose Avicel PH-105 (FMC BioPolymer, Philadelphia, PA, nominal particle size: 20 μm) were also imaged. For each sample, about 20 pieces were randomly imaged by the functionalized AFM tip at an average scanning speed of 6 μm/s. 100 recognition images in size of 1 μm × 1 μm were randomly selected for the recognition area percentage calculation.

## Abbreviations

AFM: Atomic force microscopy; P: Poplar; SG: Switchgrass; CS: Corn stover; CBM: Carbohydrate-binding module; DAP: Dilute acid pretreatment; RAP: Recognition area percentage.

## Competing interests

The authors declare that they have no competing interests.

## Authors’ contributions

BX conceived of the study. MZ performed the experiment and analyzed the data. GC designed the schemes of AFM tip chemical functionalization. RK generated the plant materials. MZ and BX drafted the manuscript. All authors read and approved the final manuscript.

## Supplementary Material

Additional file 1 Supporting figures and tables. Click here for file
