# Supplementary material for: Mapping out the structural changes of natural and pretreated plant cell wall surfaces by atomic force microscopy single molecular recognition imaging
Source: Biotechnol Biofuels. 2013 Oct 11;6:147. doi: 10.1186/1754-6834-6-147 (PMC3852143; doi:10.1186/1754-6834-6-147)
Supplement: Additional file 1 — Supporting figures and tables. [file 1754-6834-6-147-S1.pdf]

Supporting figure and tables

Mapping out the structural changes of natural and pretreated plant cell wall surfaces by atomic force microscopy single molecular recognition imaging

Mengmeng Zhang[1], Guojun Chen[1,2], Rajeev Kumar[3], Bingqian Xu[1,*]

[1]Single Molecule Study laboratory, College of Engineering and Nanoscale Science and Engineering Center, University of Georgia, Athens, GA 30602, USA

[2]Bruker Nano Surface Division, 112 Robin Hill Road, Santa Barbara, CA 93117, USA

[3]Center for Environmental Research and Technology, Bourns College of Engineering, University of California, Riverside, 1084 Columbia Avenue, Riverside, CA 92507, USA

*Corresponding author

E-mail: bxu@engr.uga.edu; Fax: +1-706-542-3804; Tel: +1-706-542-0502

1. Dilute acid pretreatment (DAP) conditions of poplar, switchgrass and corn stover

Nominations of all samples and their pretreatment conditions are listed in Table S1.

Table S1. Dilute acid pretreatment conditions of poplar, switchgrass and corn stover

Plant species	Sample ID	Solid loading (%)	Temperature (°C)	Reaction time (min)	Acid loading (g/g dry

					wt.)
Poplar	Natural-P	-	-	-	-
	0.03%P-135	5%	135	20	0.005
	0.5%P-135	5%	135	20	0.095
	1%P-135	5%	135	20	0.2
	2%P-135	5%	135	20	0.4
Switchgrass	Natural-SG	-	-	-	-
	0.03%SG-135	5%	135	20	0.005
	0.5%SG-135	5%	135	20	0.095
	1%SG-135	5%	135	20	0.2
	2%SG-135	5%	135	20	0.4
	0.5%SG-160	5%	160	20	0.095
Corn stover	Natural-CS	-	-	-	-
	0.03%CS-135	5%	135	20	0.005
	0.5%CS-135	5%	135	20	0.095
	1%CS-135	5%	135	20	0.2
	2%CS-135	5%	135	20	0.4
	0.5%CS-160	5%	160	20	0.095

P- poplar; SG- switchgrass; CS- corn stover

2. Calculation of recognition area percentage (RAP)

An example of recognition area percentage (RAP) calculation is shown in Figure S1 and equation (1-3). A 250 nm × 250 nm area was chosen to highlight the binding sites.

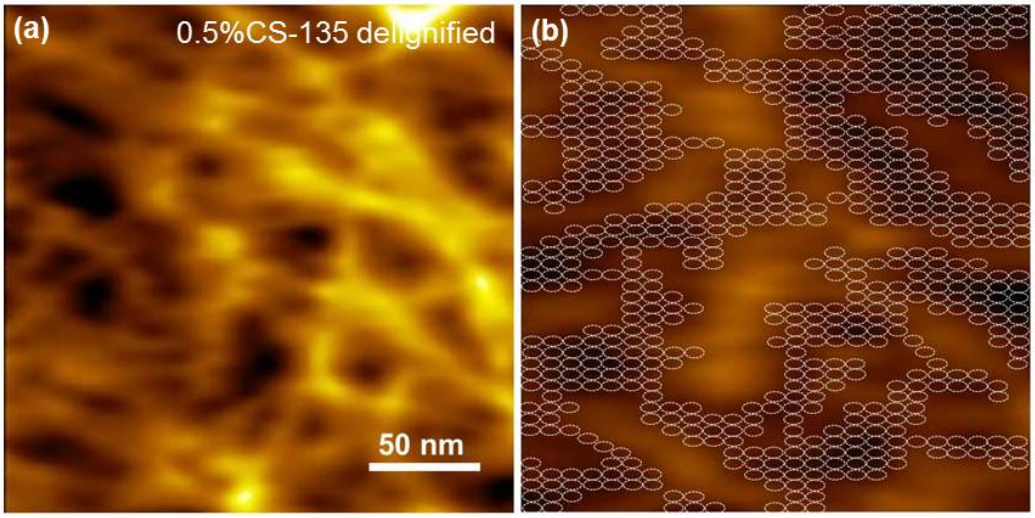

Figure S1. Example of recognition area percentage (RAP) calculation on delignified corn stover cell wall pretreated by 0.5% sulfuric acid. (a) Topography image; (b) Recognition image. The recognition binding sites are highlighted by white circles.

The RAP of a single recognition was calculated using equation (1):

$$\text{Individual RAP (\%)} = \frac{\text{Recognition area on crystalline cellulose microfibrils (nm}^2\text{)}}{\text{Total surface area of imaged plant cell wall surface (nm}^2\text{)}} \quad (1)$$

In this work a single recognition image is in size of 1 μm × 1μm.

The average RAP and total RAP were defined in equation (2) and (3):

$$\text{Average RAP (\%)} = \frac{\sum \text{Individual RAP of area N}}{\text{Number of area N}} \quad (2)$$

$$\text{Total RAP (\%)} = \frac{\sum \text{Individual RAP of area N}}{100} \quad (3)$$

Here N is the area type (A-G). The recognition area was highlighted as the white circle in the recognition image as shown in Figure S1(b). The un-recognized area was supposed to be covered by non-carbohydrate residues, exposed amorphous cellulose or partially twisted crystalline cellulose.

3. Effect of DAP on natural and delignified plant cell walls of poplar and switchgrass

3.1 Effect of DAP on natural plant cell walls of poplar and switchgrass

The surfaces of natural and 0.5% sulfuric acid pretreated poplar and switchgrass cell walls were characterized and the results are shown in Figure S2 and S3.

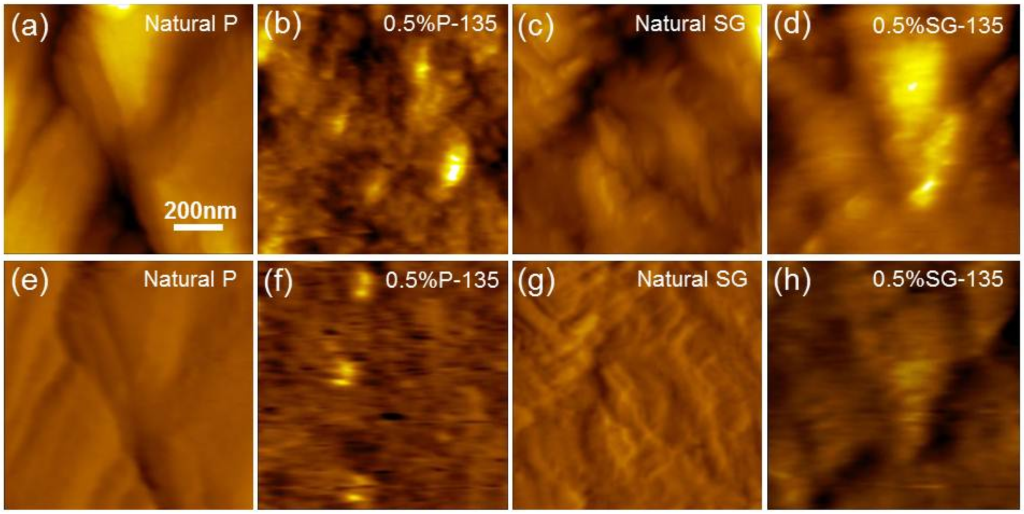

Figure S2. Topography (a-d) and recognition (e-h) images of natural and dilute acid pretreated poplar (0.5%P-135) and switchgrass (0.5%SG-135).

The size of lignin agglomerates on poplar cell wall surface was larger than that on switchgrass, showing a more intact solid structure of lignin on poplar cell walls. The RAPs of natural poplar and switchgrass was 12.9% and 13.8%, respectively. After DAP with 0.5% sulfuric acid, the RAPs of poplar and switchgrass increased to 17.4% and 20.4%, respectively.

3.2 Effect of DAP on delignified plant cell walls of poplar and switchgrass

After delignification, the fine crystalline cellulose structure was exposed and the recognition was largely improved as shown in Figure S3.

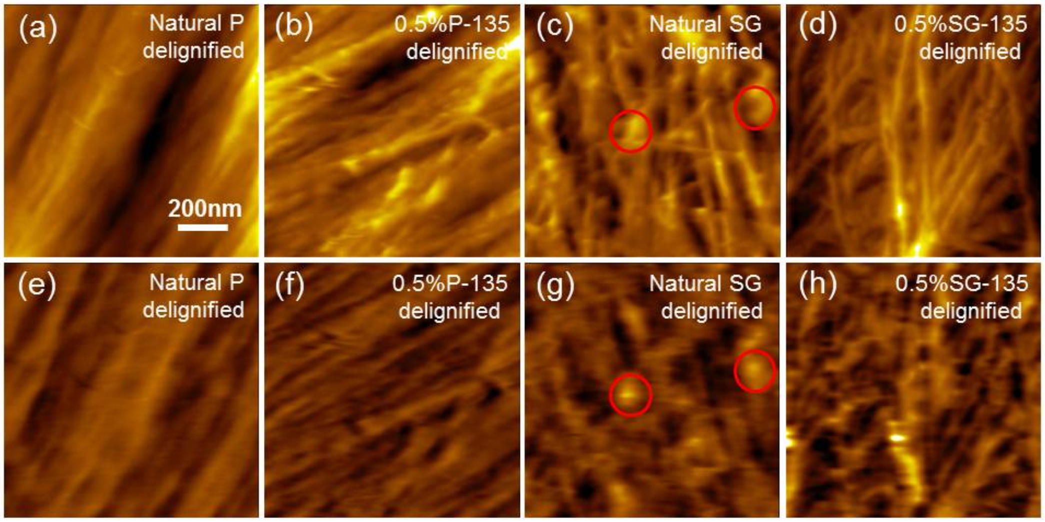

Figure S3. Topography (a-d) and recognition (e-h) images of delignified natural and DA pretreated poplar (0.5%P-135) and switchgrass (0.5%SG-135). Residual components or unfavorable position of crystalline cellulose which are not recognized are highlighted in red circles.

For both poplar and switchgrass cell walls without DAP, the surfaces after delignification were majorly composed of crystalline cellulose together with other un-removed, linking matrix components (e.g., hemicellulose). Some representative non-cellulose components which were not recognized were marked in red circles. The RAPs of delignified poplar and switchgrass without DAP were 21.7% and 26.1%, respectively. With 0.5% acid pretreatment, the RAPs of poplar and switchgrass increased to 32.3% and 33.1%. The delignified switchgrass cell wall structure showed more interwoven crystalline cellulose than those of poplar.

3.3 Effect of DAP with 0.03% and 1% sulfuric acid

The topography and recognition images of poplar and switchgrass cell walls pretreated by 0.03% and 1% sulfuric acid are shown in Figure. S4.

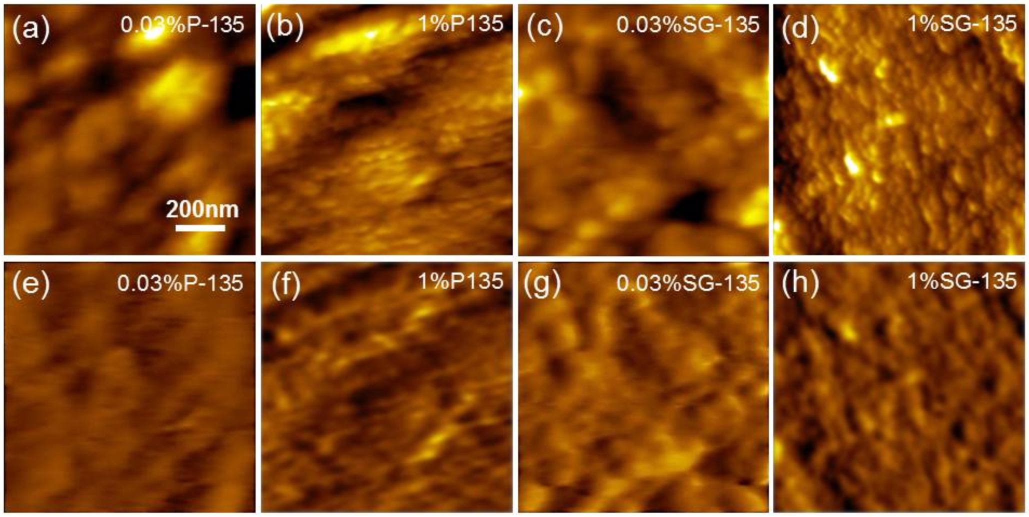

Figure S4. Topography (a-d) and recognition (e-h) images of DA pretreated poplar (0.03%P-135 and 1%P-135) and switchgrass (0.03%SG-135 and 1%SG -135).

Under higher acid concentrations, the lignin residues were smaller in size but covered the cell wall surface more evenly therefore reduced the recognition area and RAPs. The RAPs of pretreated poplar decreased from 14.8% (0.03%P-135) to 13.5% (1%P-135) and the RAPs of switchgrass decreased from 17.3% of 0.03%SG-135 to 15.2% of 1%SG-135.

3.4 Effect of DAP with 0.03% and 1% sulfuric acid after delignification

Figure S5 shows the topography and recognition images of the above pretreated poplar and switchgrass cell walls after delignification.

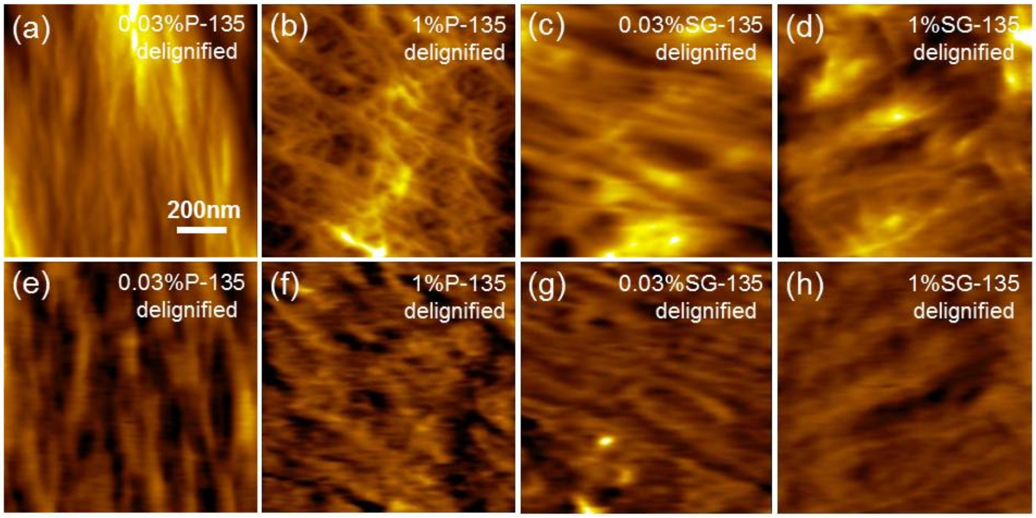

Figure S5. Topography (a-d) and recognition (e-h) images of delignified DA pretreated poplar (0.03%P-135 and 1%P-135) and switchgrass (0.03%SG-135 and 1%SG-135).

It can be clearly seen that the cell wall pretreated with higher acid concentration showed a more interwoven morphology, indicating a more efficient removal of hemicellulose. The RAPs of delignified cell walls after DAP increased from 29.4% (0.03%P-135) to 41.2% (1%P-135) for poplar and increased from 31.1% (0.03%SG-135) to 43.8% (1%SG-135) for switchgrass. This result again demonstrated a better effect of hemicellulose removal under higher acid concentration.

3.5 Effect of DAP with 2% sulfuric acid

Additionally, the cell walls of poplar, switchgrass, and corn stover pretreated by 2% acid were also characterized and the representative images are shown in Figure S6.

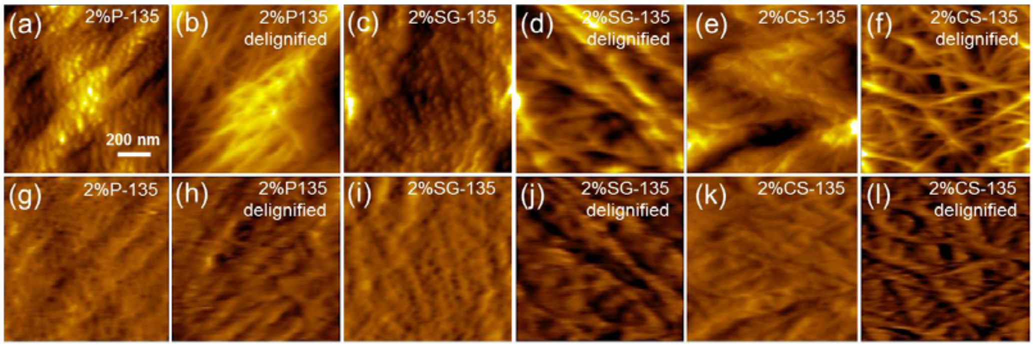

Figure S6. Topography (a-f) and recognition (g-l) images of DA pretreated and delignified poplar (2%P-135), switchgrass (2%SG-135) and corn stover (2%CS-135).

The cell walls of all species pretreated with 2% acid showed well-distributed small lignin agglomerates and cross-linking crystalline cellulose morphology after delignification. The RAPs of 2%P-135, 2%SG-135 and 2%CS-135 increased from 13.2%, 16.8% and 16.2% to 41.9%, 42.6% and 47.5%, respectively after delignification.

<u>4. Area type distributions of natural and pretreated cell walls of poplar and switchgrass</u>

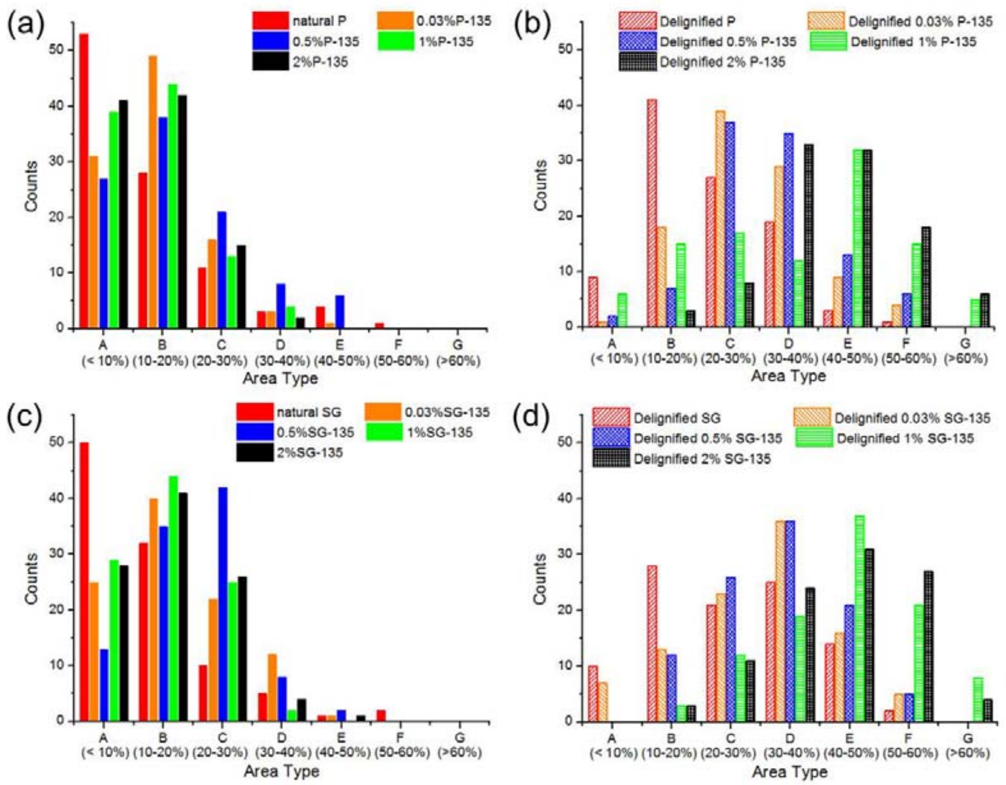

Figure S7. Area type distributions of natural (a and c) and pretreated (b and d) cell walls of poplar and switchgrass.

Figure S7 reveals diverse distributions of recognition images of natural and DA pretreated poplar and switchgrass. Similar to corn stover, for natural poplar and switchgrass, more than half of the surface morphologies were located in type A and B. After DAP with 0.03%, 0.5%, 1% and 2% dilute acid, the more cell wall surfaces showed the features in type C and D. After delignification, the surfaces of DA pretreated cell walls showed more features determined as type D through G and the majority of recognition images of natural samples were determined as type A though D.

5. Topography and recognition images and area type distributions of pure microcrystalline cellulose (Avicel)

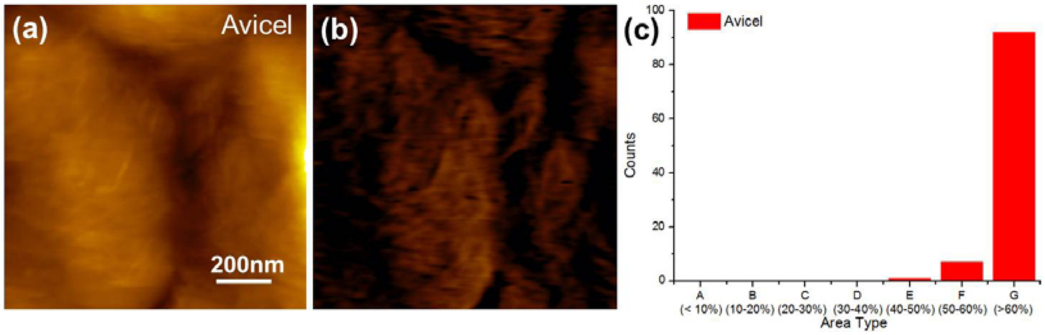

Figure S8. Topography (a) and recognition (b) images of pure microcrystalline cellulose (Avicel). The area type distribution of Avicel surfaces are shown in (c).

The surface of Avicel was full covered by short microcrystalline cellulose fibrils and over 70% of the surface components were recognized in Figure S8(b). More than 90 recognition images showed the features of type G (with RAP > 60%) as shown in Figure S8(c).

6. Chemical component changes of natural, DA pretreated and delignifed poplar cell wall surface by grazing angle attenuated total reflectance Fourier transform infrared (ATR-FTIR) spectroscopy

FTIR measurements were taken with a Nicolet Model 6700 spectrometer (Thermo Electron Corporation, Waltham, MA) with a grazing angle attenuated total reflectance accessory. Spectra were obtained with 4 cm^{-1} resolution at 64 scans for both the background and samples. After drying, the sample was pressed against the Ge hemispherical ATR crystal surface by a build-in pressure applicator (Harrick

Scientific Products, Inc. Pleasantville, NY). The representative spectra of each poplar sample are shown in Figure S9 below.

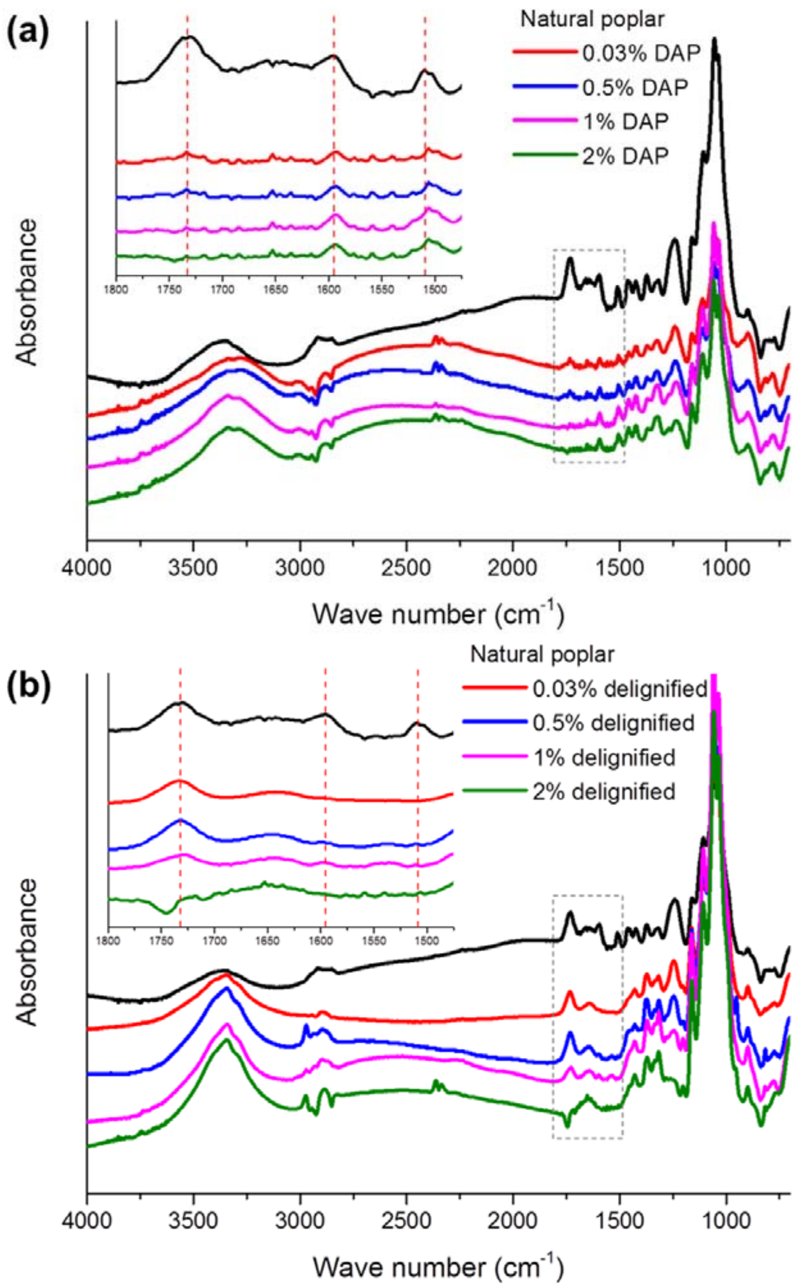

Figure S9. ATR-FTIR spectra of natural, DA pretreated (a) and deliginified (b) poplar cell wall surface.

In Figure S9(a), the ATR-FTIR spectra show the chemical component changes of dilute acid pretreated poplar cell wall surface. The inset from the dashed, grey square highlights the surface content changes of hemicellulose and lignin. The dashed, red lines highlight the positions of the bands at 1732 cm^{-1} (ascribed to carbonyl group, indicating hydrolysis of hemicellulose and cleavage of lignin side chains), 1595 cm^{-1} and 1510 cm^{-1} (ascribed to aromatic skeletal vibrations of lignin)[1-3]. The spectra showed an obvious decrease of hemicellulose and slight decrease of lignin in peaks. For sample pretreated by 1% and 2% acid, the surface content of lignin started to slightly increase, indicating a possibility of more extensive surface coverage of lignin which agreed with the AFM recognition result.

After delignification, the lignin on the cell wall surface almost disappeared and the decrease of hemicellulose under higher acid concentration is more pronounced as previously determined by RAP result (Figure S9 (b)).

7. Effect of dilute acid pretreatment of switchgrass under different temperatures

The topography and recognition images of switchgrass pretreated by 0.5% acid under 135°C and 160°C are shown in Figure S10.

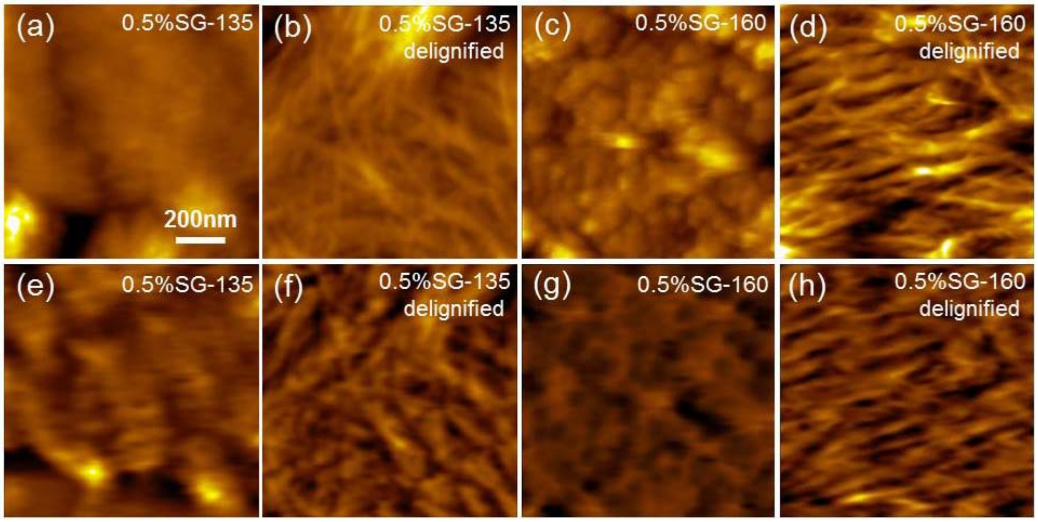

Figure S10. Topography (a-d) and recognition (e-h) images of DA pretreated delignified switchgrass (0.5%SG-135 and 0.5%SG-160).

Similar to corn stover, the re-precipitated lignin agglomerates on surface of DA pretreated switchgrass decreased under higher reaction temperature and the RAPs also increased slightly from 27.5% to 28.5% (after calibration, the same for the following RAPs). After removal of lignin, the crystalline cellulose underneath showed more fine structure and was better separated under higher pretreatment temperature with the increase of RAP from 44.5% to 47.8%.

References:

1. Kumar R, Mago G, Balan V, Wyman CE: **Physical and chemical characterizations of corn stover and poplar solids resulting from leading pretreatment technologies.** *Bioresour Technol* 2009, **100:**3948-3962.

2. Kristensen JB, Thygesen LG, Felby C, Jorgensen H, Elder T: **Cell-wall**

structural changes in wheat straw pretreated for bioethanol production. *Biotechnol Biofuels* 2008, **1:**5.

3. Chundawat SPS, Venkatesh B, Dale BE: **Effect of particle size based separation of milled corn stover on AFEX pretreatment and enzymatic digestibility.** *Biotechnol Bioeng* 2007, **96:**219-231.
